# Supplementary material for: Cow Milk Extracellular Vesicle Effects on an In Vitro Model of Intestinal Inflammation
Source: Biomedicines. 2022 Feb 28;10(3):570. doi: 10.3390/biomedicines10030570 (PMC8945533; doi:10.3390/biomedicines10030570)
Supplement: Supplementary file 1 [file biomedicines-10-00570-s001.zip › biomedicines-1577274-supplementary.pdf]

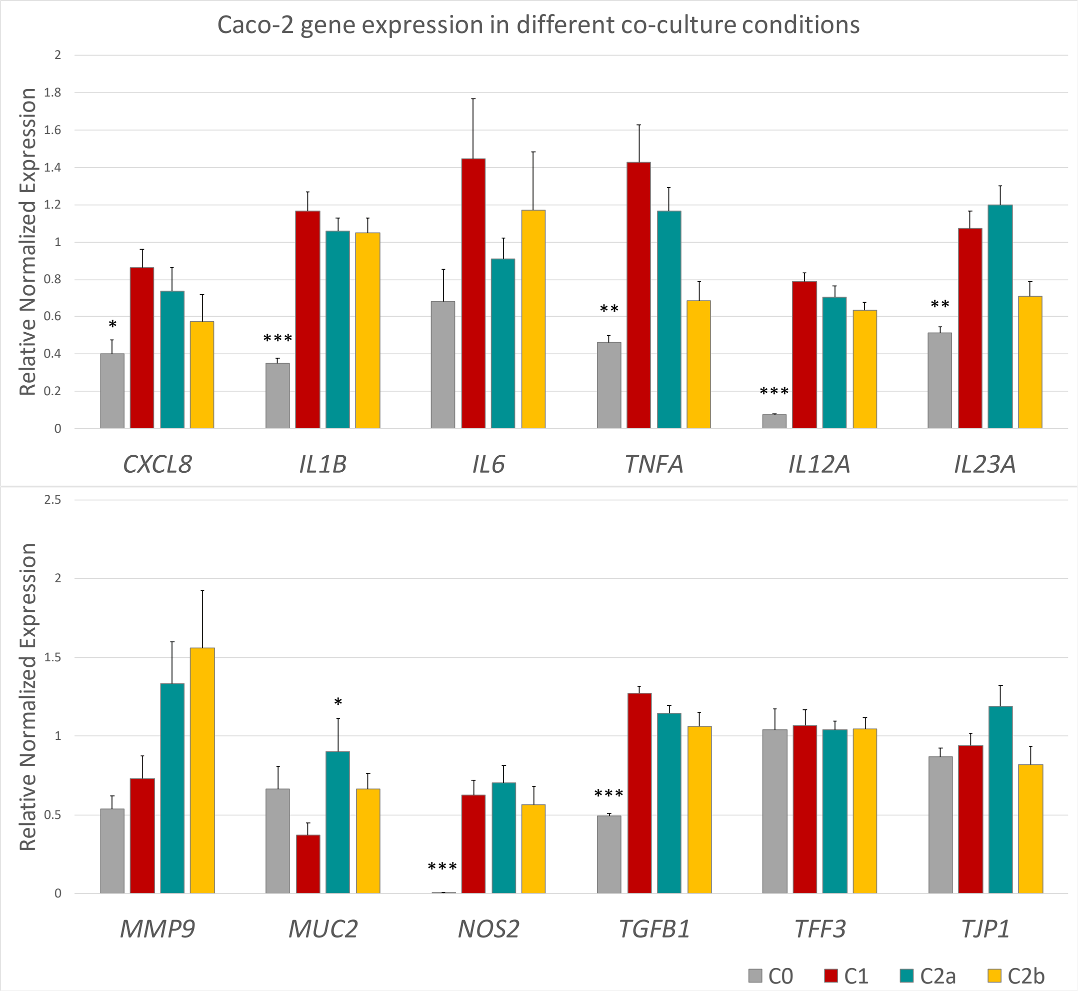

Figure S1. Histogram of Caco-2 tested gene expression in the different culture conditions: Co-culture of Caco-2 and THP-1 cells in basal conditions (C0 - grey), Inflamed co-culture (C1 - red), 10^8 mEV administering to Caco-2 cells in inflamed co-culture (C2a - green) and 10^8 mEV administering to Caco-2 and THP-1 cells in inflamed co-culture (C2b - yellow). Differences (others *vs* C1) were evaluated through the Kruskal-Wallis test and applying the post-doc Dunn's Multiple Comparison Test. The asterisks indicate the statistical significance: * p<0.05, ** p<0.01 and *** p<0.001.

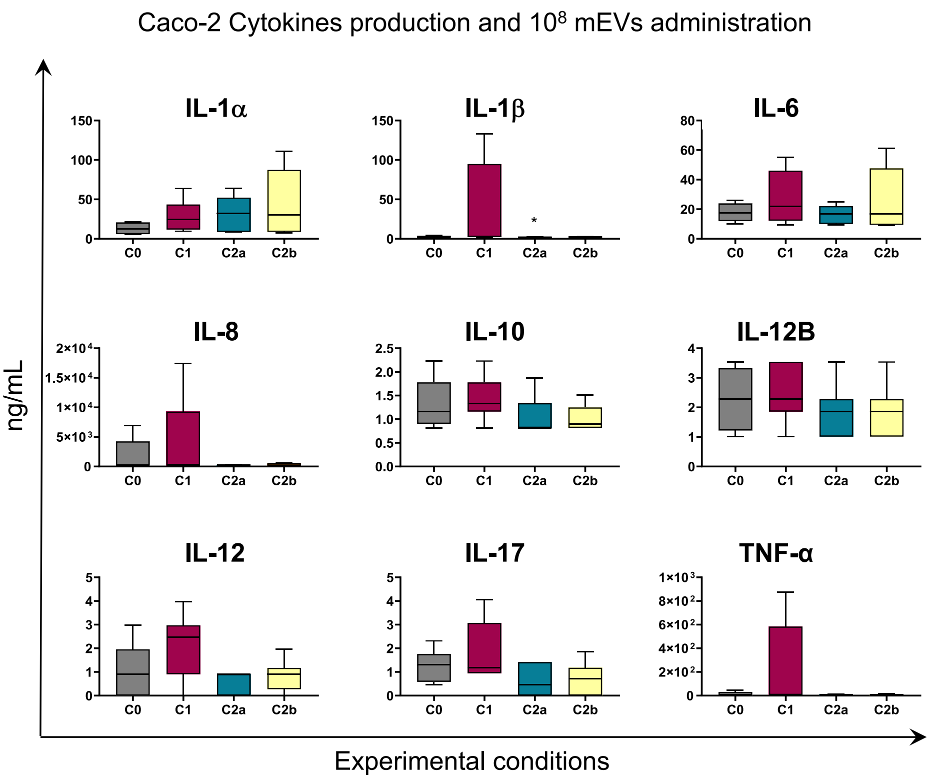

Figure S2. 10^8 mEV impact on cytokine production by Caco-2. Co-culture of Caco-2 and THP-1 cells were left untreated (basal condition, C0), or stimulated with IFN-γ and LPS (inflamed co-culture, C1). 24h post-stimulation, 10^8 mEV suspension was added to Caco-2 cells in inflamed co-culture (C2a) or to both Caco-2 and THP-1 cells in inflamed co-culture (C2b). 24h later, culture supernatants were collected, and levels of cytokines were determined through ELISA. Date are presented as box-and-whisker plots displaying median and interquartile range (boxes) and minimum and maximum values (whiskers). Values of C0, C2a, C2b were compared to C1, using a ANOVA followed by Dunnett's multiple comparison test or a Kruskal-Wallis test followed by Dunn's multiple comparison test; * p<0.05, ** p<0.01 and *** p<0.001.

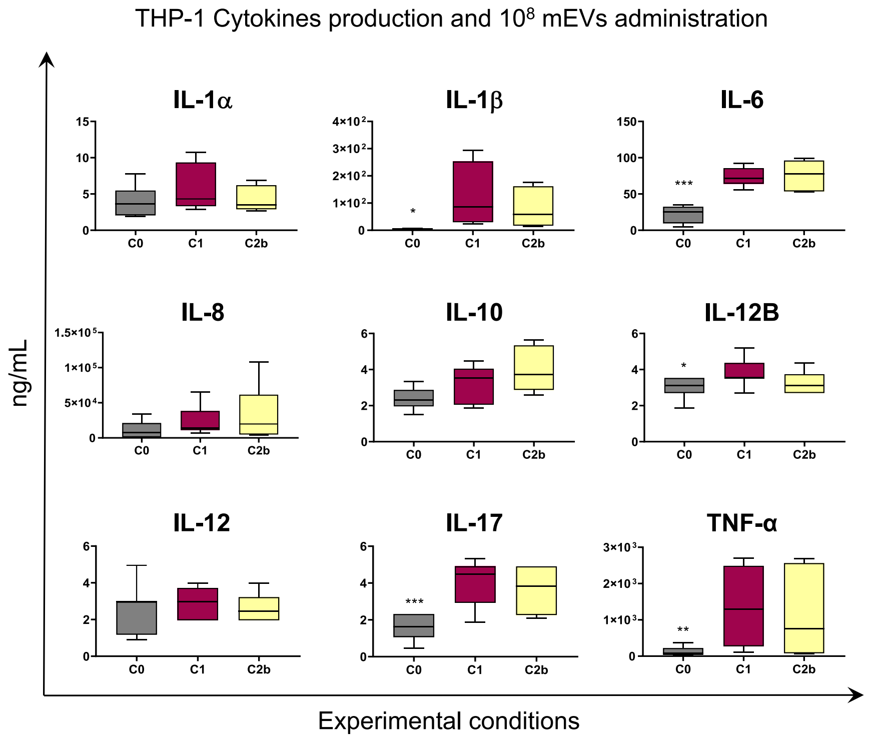

Figure S3. 10^8 mEV impact on cytokine production by THP-1**.** Co-culture of Caco-2 and THP-1 cells were left untreated (basal condition, C0), or stimulated with IFN-γ and LPS (inflamed co-culture, C1). 24h post-stimulation, 10^8 mEV suspension was added to both Caco-2 and THP-1 cells in inflamed co-culture (C2b). 24h later, culture supernatants were collected, and levels of cytokines were determined through ELISA. Date are presented as box-and-whisker plots displaying median and interquartile range
(boxes) and minimum and maximum values (whiskers). Values of C0, C2a, C2b were compared to C1, using a ANOVA followed by Dunnett's multiple comparison test or a Kruskal-Wallis test followed by Dunn's multiple comparison test; * p<0.05, ** p<0.01 and *** p<0.001.
